# Using the Scanning Fluid Dynamic Gauging Device to Understand the Cleaning of Baked Lard Soiling Layers

**DOI:** 10.1007/s11743-015-1737-z

**Published:** 2015-10-10

**Authors:** Akin Ali, Zayeed Alam, Glenn Ward, D. Ian Wilson

**Affiliations:** Department of Chemical Engineering and Biotechnology, New Museums Site, Pembroke Street, Cambridge, CB2 3RA UK; Procter & Gamble Technical Centres Ltd, Whitley Road, Longbenton, Newcastle-upon-Tyne, NE12 9TS UK

**Keywords:** Adhesion, Cohesive strength, Cleaning, Fouling, Fats

## Abstract

Extended or repeated heating of food fats promotes polymerisation reactions that produce difficult-to-remove soil layers. Cleaning of these baked-on/burnt-on fat deposits was investigated using model layers generated by baking lard on 316 stainless steel discs. Rigorous characterisation of the layer material was difficult, as it was insoluble in most solvents. Cleaning was studied using the scanning fluid dynamic gauging technique developed by Gordon et al. (Meas Sci Technol 21:85–103, 2010), which provides non-contact in situ measurement of layer thickness at several sites on a sample in real time. Tests at 50 $^\circ$C with alkali (sodium hydroxide, pH 10.4–11) and three surfactant solutions indicated two removal mechanisms, related to the (1) roll-up and (2) dispersion mechanisms reported for oily oils, namely (1) penetration of solvent at the soil–liquid interface, resulting in detachment of the soil layer as a coherent film, observed with linear alkylbenzene sulfonic acid (LAS) and Triton X-100 and aqueous sodium hydroxide at pH 10.4–11; and (2) the breakdown promoted by the agent penetrating through the layer, observed with cetyl trimethyl ammonium bromide (CTAB), in which CTAB antagonised the cleaning action of LAS.

## Introduction

The problem of cleaning difficult-to-remove food soils is ubiquitous in the food industry. During cooking, fats and oils undergo a series of polymerisation reactions that can leave undesirable, unwanted deposits on food processing surfaces such as baking trays and frying pans. This accumulation of material generated by thermal processing is called *fouling*, and the deposits formed are known as ‘soils’.

### Autoxidation Fouling

Fouling is defined as the unwanted accumulation of material on and/or into process surfaces. Any deposit that needs to be cleaned is initially generated by a fouling step. Understanding how a deposit forms can yield insight into how best to remove it and how to prevent its formation.

The polymerisation of food lipids occurs via autoxidation, which is the autocatalytic oxidation of hydrocarbons. Autoxidation proceeds via a free radical chain mechanism initiated at points of unsaturation within the lipid. Extensive research has been carried out to understand the mechanisms involved in the autoxidation of unsaturated hydrocarbons and organic chemicals. The reaction scheme widely used is described in the review by Watkinson and Wilson [1].

The primary oxidation products of carbon double bonds are peroxides and polyperoxides. Autoxidation is an endothermic process, with a high activation energy, and is thought to be the process which converts fats into the insoluble, difficult-to-clean deposits that are the focus of this study. It occurs slowly at ambient temperatures, but during baking, where the temperatures can reach 250 $^\circ$C, it occurs more quickly. Ultraviolet light, heat, metal ions or chemical initiators catalyse the initiation steps. The formation of polyperoxides leads to the formation of gums. Longer gum molecules are insoluble and precipitate (from solution) before adhering to the substrate surface.

Food lipids also contain a variety of glycerides and fatty acids. Common food soils also contain water, salts, starches and proteins, all of which contribute to physical and chemical changes which occur during cooking. Food lipid autoxidation therefore produces complex mixtures containing a range of chemical species. Unbaked lipids themselves often present a difficult cleaning challenge, particularly when they have aged on the surface. They typically require hot water and some degree of detergent or lipase enzyme to achieve sufficient cleaning [2]. In this study, lard was chosen as a model foodstuff representative of food lipids. On baking, lard forms difficult-to-remove polymerised deposits.

### Cleaning Agents

The earliest reports of cleaning agents for household use (such as soaps) date back to 2200 bc [3]. These were the first alkaline water-based soaps, and remained largely unchanged until the early part of the twentieth century, when synthetic detergents were produced on a large scale [4]. Modern cleaning agents are now complex mixtures comprising surfactants, enzymes and chelants (i.e. sequestrants). Cleaning agents may also contain alkaline agents to control pH; anti-redeposition agents, to prevent removed soil from re-attaching to cleaned surfaces; suds control agents, to inhibit foam formation; corrosion inhibitors, such as sodium silicate, to prevent damage to dishwashers; and bleach, to abate opaque stains. They also contain a number of ingredients that do not directly aid cleaning performance, but are used to enhance the user experience, such as colorants, opacifiers and fragrances [3, 5].

#### Soil Removal by Surfactants

Broadly speaking, removal can be classified as follows:

1. *Dissolution* of foulant into solution. This occurs when the cleaning agent is a solvent for the material in the deposit. Certain soils may contain soluble and insoluble components. Dissolution of some of the deposits allows the ingress of cleaning agent into the deposit, and this can promote breakdown of the deposit layer.
2. *Mechanical* removal occurs when the force imposed on the deposit by cleaning implements (such as a sponge) or the flow of cleaning solution is sufficient to deform and disrupt the layer. This can cause either adhesive removal, where soil–substrate bonds are broken, or cohesive removal, where soil–soil bonds are broken. An example of mechanical cleaning is the removal of soft solid deposits by impinging liquid jets [6].
3. *Chemical* cleaning occurs when cleaning agents such as enzymes sever chemical bonds, promoting cohesive and/or adhesive removal. Alternatively, chemical agents can be used to change the soil into a more removable form. For example, Jurado et al. [2] used lipase enzymes to hydrolyse the ester bonds to aid in the cleaning of triolein and tributyrin oils.

**Fig. 1:**
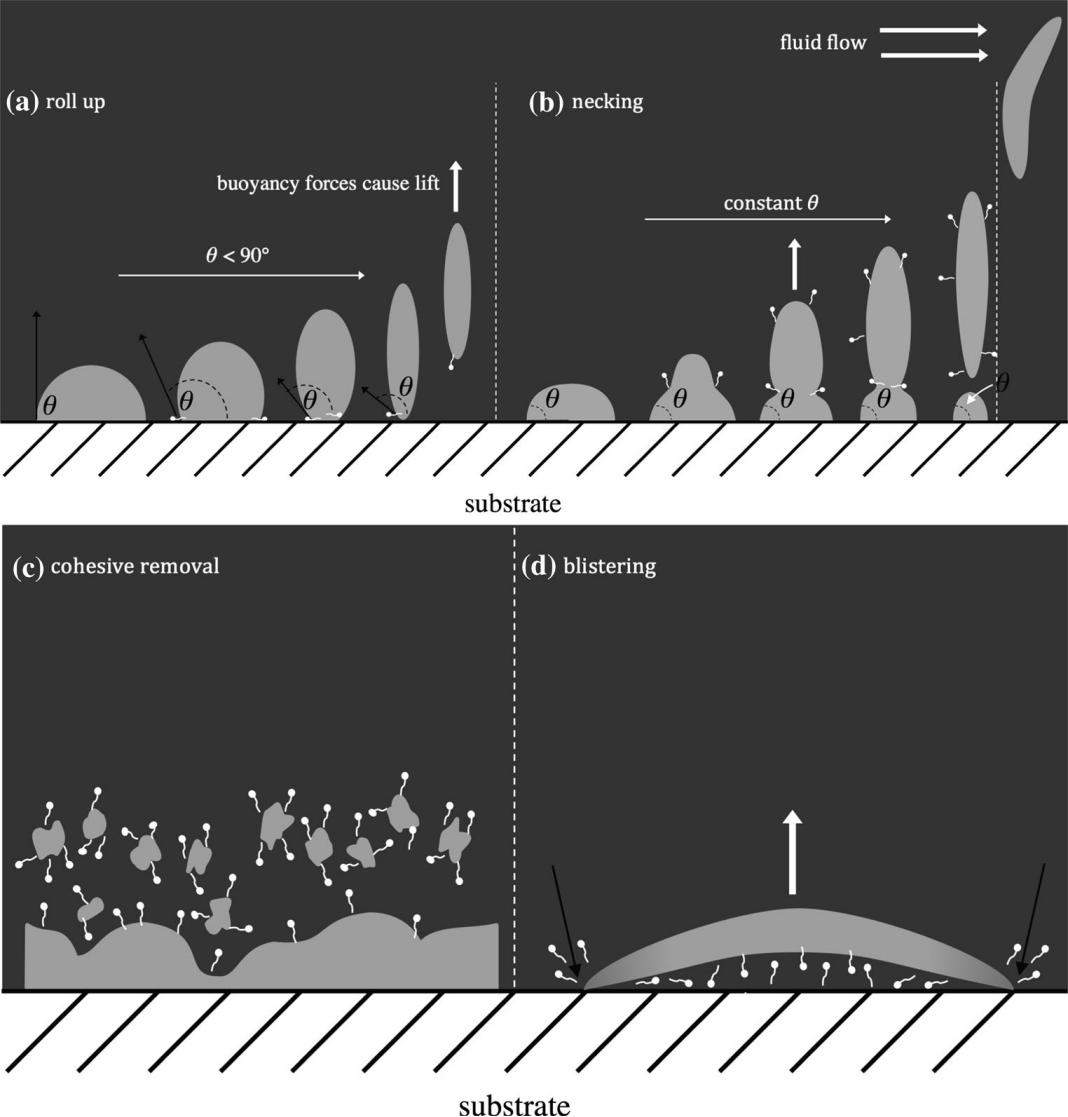
Schematic of **a** roll-up and **b** necking for oil soils. Oil droplets are attached to a substrate whilst immersed in a cleaning agent. Schematic of removal for solid lipid soils immersed in cleaning solution for **c** cohesive removal and **d** blistering

Adhesive and cohesive failure can occur simultaneously. Surfactants may promote adhesive and/or cohesive removal. During the former, surfactants locate themselves at the soil–substrate interface and promote an increase in the soil–substrate equilibrium contact angle, $\theta$, as illustrated in Fig. 1a. When $\theta$ reaches 180$^\circ$, the soil rolls up, and buoyancy forces promote soil detachment. This process is accordingly labelled ‘roll-up’. If $\theta$ reaches an equilibrium value that lies between 90$^\circ$ and 180$^\circ$, part of the soil then remains on the surface, and surfactants promote (cohesive) rupture of oil droplets. This process is called ‘necking’ and is illustrated in Fig. 1b. In the case of necking, additional (i.e. mechanical) forces are required for complete removal. For oily soils, removal often occurs via ‘roll-up’ or ‘necking’ [7].

For solid soils, removal requires penetration of surfactants (and associated water molecules) to liquefy the soil so that roll-up can occur [7–9]. When the surfactant cannot penetrate the surface, an increase in temperature may be required to aid liquefaction (i.e. by melting). Moreover, surfactants may aid absorption of other components of the cleaning agent, and this can also improve cleaning.

In this study, we have identified two removal mechanisms for solidified lipid soils, which are shown in Fig. 1c and d, and are analogous to necking and roll-up removal mechanisms for oily soils. Due to the structure of polymerised soils, it is more difficult for surfactants to absorb into (or liquefy) the deposit, and as a result, the removal mechanisms are different. In certain cases, such as that in Fig. 1c, the surfactant may penetrate the soil at the soil–liquid interface and promote cohesive removal of debris; this is analogous to necking.

Surfactants can also promote adhesive removal of the baked lipids, whereby ingress of the surfactant occurs at the soil–substrate interface. This weakens soil attachment to the substrate such that buoyancy forces are sufficient to lift the deposit from the substrate. This process is described as blistering (see Fig. 1d). Surfactants may also play a role in preventing re-attachment, and any liquid flow may carry the removed deposit away from the substrate.

 To our knowledge, the study by Dunstan and Fletcher [10] is the only one in the published literature to have investigated the cleaning of thermally treated fats. The authors prepared greasy soil samples comprising an equal weight mixture of lard, vegetable oil and shortening, and looked at the effects of cationic, anionic and nonionic surfactants on cleaning. Their soils were tacky, semi-solid materials, and did not cross the hardening threshold experienced by the soils used in this study. As such, their soils presented a cleaning challenge different from the ones investigated in this study, and their findings are not directly transferrable.

### Solubility Parameter

Polymerised oil and grease soils are marked by a poor tendency to dissolve in aqueous-based cleaning agents. This tendency, or aversion, can be quantified in terms of the solubility parameter, $\delta _{{i}}$. This provides insight into the type of molecular interactions that a given species may engage in. Hildebrand and Scott [11] defined the solubility parameter as the energy required to break all intermolecular bonds per unit volume:

1 $$\begin{aligned} \delta _{{i}}= \sqrt{\frac{\Delta H_{\mathrm {vap}}}{V_{\mathrm {m}}}} \end{aligned}$$

where $\delta _{{i}}$ is the solubility parameter of species *i*, $\Delta H_{\mathrm {vap}}$ is the enthalpy of vapourisation (i.e. the energy required to break all the liquid-liquid bonds) and $V_{\mathrm{m}}$ is the average molar volume.

Hansen et al. [12] split the solubility parameter into three components, labelled dispersive ($\delta _{\mathrm {d}}$), polar ($\delta _{\mathrm {p}}$), and hydrogen bonding ($\delta _{\mathrm {h}}$). Each of these can be considered one dimension of a three-dimensional solubility parameter, such that

2 $$\begin{aligned} \delta _{{i}}^2 = {\delta _{\mathrm {d}}}^2 + {\delta _{\mathrm {p}}}^2 + {\delta _{\mathrm {h}}}^2 \end{aligned}$$

The $\delta _{\mathrm {d}}$, $\delta _{\mathrm {p}}$, $\delta _{\mathrm {h}}$ values, which are also temperature-dependent, are collectively known as the Hansen solubility parameter (HSP) for a particular species. Hansen solubility parameters have found widespread use in a range of academic and industrial studies, including thermodynamic models for polymer solutions, characterisation of the performance and nature of surface coatings, analysis of proteins, and evaluation of the uptake of chemicals through coatings [13].

### Fluid Dynamic Gauging

Studying soft solid layers immersed in liquid can be challenging, as removing them in the liquid for analysis can modify their structure or state. Fluid dynamic gauging (FDG) is a non-contact measurement technique developed to study the fouling and cleaning of soft fouling layers immersed in opaque liquid environments. Measurements of deposit thickness and strength are made in situ and in real time. Inspired by pneumatic gauging [14], FDG instead uses a liquid flow to make its measurements. It is therefore a technique that can readily be used to test the effectiveness of cleaning formulations and to quantify any subsequent breakdown.

**Fig. 2:**
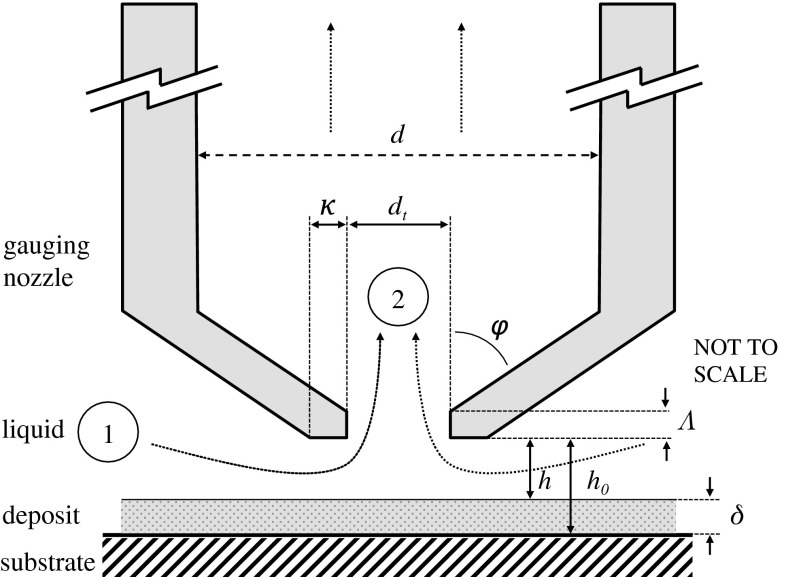
Schematic of the action in an FDG gauging nozzle. *Dotted lines* denote liquid flow path in suction mode. Stations (*1*) and (*2*) indicate the region over which the majority of the pressure drop across the nozzle is generated. *Symbols* $\delta$ thickness of deposit layer; *h* clearance between nozzle and deposit layer; $h_{\mathrm {o}}$ clearance between gauging nozzle and substrate surface; $d_{\mathrm {t}}$ inner diameter of nozzle; $\kappa$ lip width; $\varphi$ nozzle angle; *d* inner tube diameter; $\Lambda$ nozzle entry length

The principle of FDG is illustrated in Fig. 2. A nozzle is located at a distance *h* from a solid surface immersed in liquid. A pressure drop, $\Delta P_{12}$, is imposed across the nozzle, causing liquid to flow into the nozzle at a mass flow rate, $\dot{m}$. In addition to *h* and $\Delta P_{12}$, the mass flow rate is sensitive to the nozzle throat diameter, $d_{\mathrm {t}}$; the nozzle lip width, $\kappa$; the liquid density, $\rho$; and viscosity, $\mu$. A dimensional analysis (see [15]) gives:

3 $$\begin{aligned} C_{\mathrm {d}} = f\left( \frac{h}{d_{\mathrm {t}}},\frac{\kappa }{d_{\mathrm {t}}}, \textit{Re}_{\mathrm {t}}\right) \end{aligned}$$

where $Re_{\mathrm {t}}$ is the Reynolds number evaluated at the throat of the nozzle (see Fig. 2), and $C_{\mathrm {d}}$ is the discharge coefficient, which is the ratio of $\dot{m}$ to the ideal mass flow rate through the nozzle and accounts for the energy losses associated with flow through the nozzle:

4 $$\begin{aligned} C_{\mathrm {d}} = \frac{{\mathrm {actual\,mass\,flow\,rate}}}{{\mathrm {ideal\,mass\,flow\,rate}}} = \frac{\dot{m}}{\tfrac{\pi }{4}d_{\mathrm {t}}\sqrt{2 \rho \Delta P_{12}}} \end{aligned}$$

**Fig. 3:**
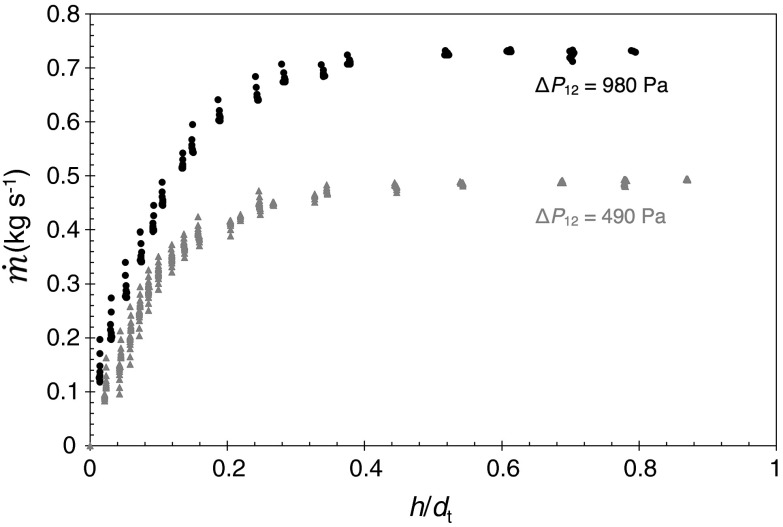
Typical ${\dot{m}}$ vs $h_{\mathrm {t}}$ FDG calibration curve sFDG device (water 20 $^\circ$C) with two different siphon driving pressures: $d_{\mathrm {t}} = 1$ mm, $\kappa = 0.5$ mm, $\phi =45^\circ$

The flow rate at a given pressure drop is very sensitive to the clearance, *h*; and knowledge of the $$C_{\mathrm {d}} - h/d_{\mathrm {t}}$$ relationship for a given $$\textit{Re}_{\mathrm {t}}$$$$(\kappa/d_{\mathrm {t}})$$ being fixed by the geometry therefore allows the distance *h* to be determined. A typical $$C_{\mathrm {d}} - h/d_{\mathrm {t}}$$ curve is shown in Fig. 3. This knowledge of *h*, and an alternative method of measuring $$h_{\mathrm {o}}$$ (e.g. by an inductance sensor of precise record of the nozzle location), allows the thickness of the fouling layer, $$\delta $$, to be calculated from:

5 $$\begin{aligned} \delta = h_{\mathrm {o}} - h \end{aligned}$$

The clearance between the gauging nozzle and substrate, $h_{\mathrm {o}}$, is measured independently or when the substrate is clean. The primary requirement for the technique is that the surface being gauged remains stiff over the duration of the test. Therefore, the gauging conditions ($\dot{m}$, $\mathrm {\Delta }P_{12}$, $d_{\mathrm {t}}$ and $\kappa$) must be set so that the shear stress exerted on the layer by the gauging flow does not deform or destroy the layer.

The scanning fluid dynamic gauge (sFDG) is an automated FDG device with computer-controlled movements, and uses a feedback loop to guide the nozzle to track the layer during swelling and cleaning at several locations [16]. The sFDG device has accuracy of $\pm$15 $\upmu$m and can operate at solution temperatures of up to 60 $^\circ$C [16]. The device can also study cleaning in situations where the substrate temperature is different from that of the bulk fluid. In the tests reported here, the liquid and soil were at the same temperature.

### Contact Angle Measurements

Contact angle measurements are made to quantify the interaction between liquids and solid (or soft solid) surfaces. The majority of tests reported here were performed on stainless steel substrates. A small number of tests employed glass surfaces with different surface properties. Surface energy measurements were made using the approach described by van Oss et al. [17], such that:

6 $$\begin{aligned} \gamma _{ij}=\gamma _{ij}^{\mathrm{AB}} + \gamma _{ij}^{\mathrm{LW}} \end{aligned}$$

where $\gamma _{ij}$ is the surface energy between phases *i* and *j* (where L, S and V are used to denote liquid, solid and vapour, respectively). $\gamma _{ij}^{\mathrm {LW}}$ is the Lifshitz–van der Waals component of surface energy. The acid–base component of surface energy, $\gamma _{ij}^{\mathrm {AB}}$, is split such that:

7 $$\begin{aligned} \gamma _{ij}^{\mathrm{AB}} = 2 \sqrt{\gamma _{ij}^- \gamma _{ij}^+} \end{aligned}$$

where $\gamma _{ij}^+$ and $\gamma _{ij}^-$ are the electron acceptor and donor components of the surface energy, respectively.

## Materials and Methods

### Sample and Substrate Preparation

Lard is a food fat regularly used in baking. As it is deficient in natural antioxidants [18], butylated hydroxytoluene (BHT) is added to commercial lard to delay the onset of rancidification. Lard was purchased in one large (6 kg) batch to reduce the effects of sample-to-sample variation. It contains 56 wt% unsaturated fat (as recorded by the supplier). Prior to testing, the lard was stored in a freezer at −4 °C.

**Fig. 4:**
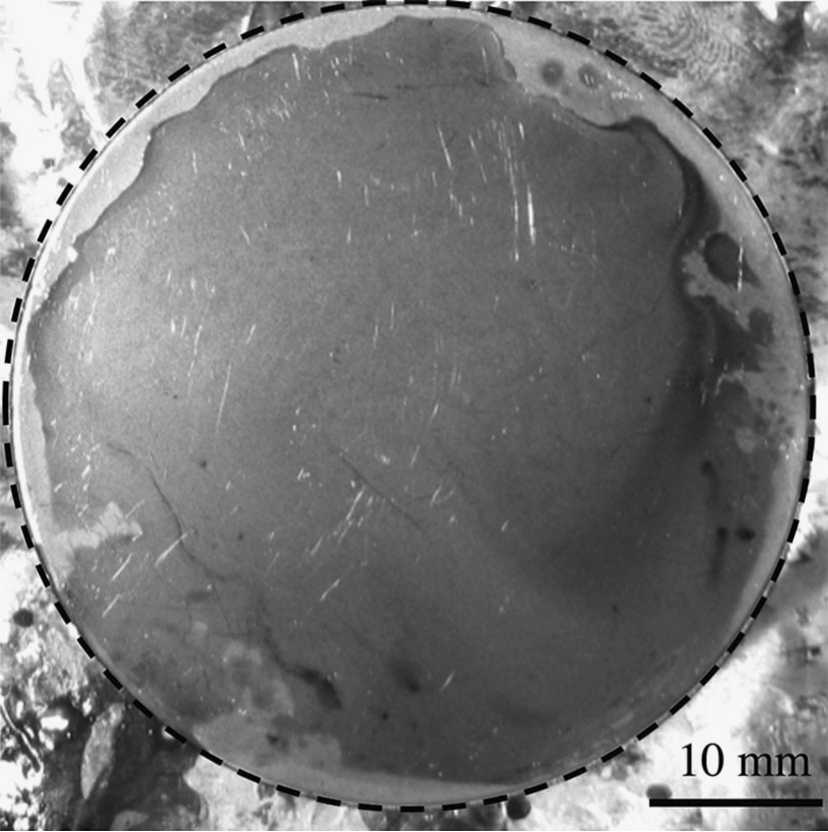
Photograph of a lard layer baked for 1.5 h at 200 $^\circ$C on 316 SS discs. The *dashed line* shows the edge of the disc

Lard samples were prepared by melting the lard at 40 $^\circ$C and then pipetting 0.1 mL of the liquid aliquot onto 50-mm stainless steel (SS) 316L discs; this resulted in baked layers that were 15–60 $\upmu$m thick (as measured by the sFDG device). The molten lard did not readily wet the SS surface, instead contracting on the substrate (see Fig. 4). This made the layers hard to prepare reproducibly, and therefore, layers were baked in batches of 12 and unsuitable samples discarded. The layers were baked for 90 min at 200 $^\circ$C. Lower temperatures and shorter baking times gave sticky, soft solid layers. Stainless steel was used as a substrate to replicate a common food processing surface. The SS disc surfaces were cleaned by immersion in 1 M sodium hydroxide solution overnight and sonication in reverse osmosis water at 40 $^\circ$C for 30 min, followed by drying in air at ambient conditions.

### Solvent Solubility Studies

Baked lipid deposits were immersed in 100 mL of solvent and left in a fume hood in a sealed vessel under agitation on a shaker plate (M802 Suspension Mixer, QM Solutions, Runcorn, UK). The aim of the solvent solubility studies was to characterise the type of interactions that the baked lard layers engaged in rather than to find the HSP values for each layer. Therefore, tests employed 20 probe liquids (instead of the 40–50 required to find $\delta _{\mathrm {d}}$, $\delta _{\mathrm {p}}$, and $\delta _{\mathrm {h}}$). The solvents used and their corresponding HSP values are presented in Table 4. Sulphuric acid and sodium hydroxide solutions, neither of which have HSP values, were also used as probe interactions with acidic and alkaline solutions. Unbaked lard, heated to 37 $^\circ$C in order to melt, was also used as a solvent. The HSP values quoted in Table 4 are for a different lard, reported by Hansen [13].

Each plate was weighed prior to the addition of lard. The mass of the baked lard was then measured prior to immersion. Once the test was complete, the sample was removed from the solvent and excess liquid was drained off. At this point, the mass was measured, and this was labelled a ‘wet’ sample. It was used as a guide to the amount of liquid absorbed into the layer during immersion. It took approximately 8 h for the layers to reach a constant weight when left in an extraction oven (110 $^\circ$C, ambient pressure, Carbolite$^\circledR$ oven). Therefore, layers were left overnight before re-weighing, and the mass recorded was then labelled the ‘dried’ weight. The difference between the initial mass after baking (and prior to immersion) and the ‘dried’ weight is the amount of baked lard removed during testing, either through dissolution or other means. Each test was repeated at least once.

### Selection of Cleaning Agents

Table 1 lists the components of the cleaning agents used in these tests. The anionic surfactant, LAS, was chosen, as it is commonly used in commercial detergent formulations. An LAS concentration of 0.88 g L$^{-1}$ was used for comparison with commercial detergents. Additionally, a cationic surfactant, (CTAB, Calbiochem$^\circledR$; Merck Millipore, Darmstadt, Germany), and a nonionic surfactant, Triton X-100 (TX-100) (Sigma-Aldrich$^\circledR$, St. Louis, MO, USA) were used during cleaning tests. As both are commonly used surfactants, there is information on their properties available in the literature. TX-100 and CTAB concentrations above the critical micelle concentration (CMC) were employed to prevent surfactant depletion affecting the cleaning experiments. Higher concentrations were not used, as micelle formation was not expected to affect removal behaviour. Solution pH was adjust using sodium hydroxide.

**Table 1:** Summary of components used in cleaning agents

Component	Type	CMC (g L$^{-1}$)	Molecular mass (g mol$^{-1}$)	Dissociation pH$^{\mathrm{a}}$	Test concentration		References
					(g L$^{-1}$)	(mmol L$^{-1}$)	
Water	Solvent	–	18	14.16	–	–	[19]
LAS	Anionic surfactant	0.100	$\sim$340	2.142	0.88	2.59	[20], Procter & Gamble
TX-100	Nonionic surfactant	$\sim$0.0131	647	n/a	0.125	0.19	[21]
CTAB	Cationic surfactant	$\sim$0.334	364.45	2.9–3.9	0.364	0.79	[22]
Fairy liquid	Commercial detergent	–	n/a	n/a	1 tablet$^{\mathrm{b}}$		Procter & Gamble

$$^{\mathrm{a}}$$ At 25 °C

$$^{\mathrm{b}}$$ Per 7 L wash

Fairy Liquid (Procter & Gamble, Weybridge, Surrey, UK) was used as a reference commercial detergent product. The effect of pH was also evaluated for the three main surfactants: LAS, CTAB and TX-100. All cleaning agents were tested under standard dishwashing conditions, i.e. with water as the solvent at pH 10.4 and 50 $^\circ$C.

### The Scanning Fluid Dynamic Gauging (sFDG) Device

**Fig. 5:**
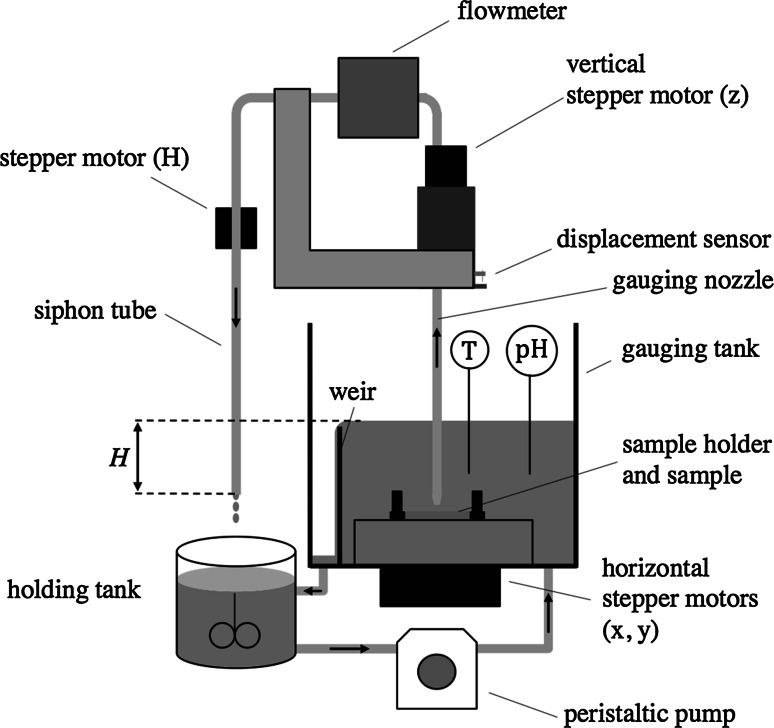
Schematic of the sFDG system. *Shaded lines* denote stainless steel tubing and *arrows* show direction of flow. *H* is the siphon hydrostatic head and is controlled by the stepper motor (*H*). The *vertical position* of the gauging nozzle is controlled by the stepper motor (*z*)

The scanning fluid dynamic gauge system (see Fig. 5) was developed by Gordon et al. [16]. The sFDG device collects sample thickness data continuously, to an accuracy of $\pm$15 $\upmu$m. A schematic of the sFDG system is shown in Fig. 5. The sample is located on a horizontal holder in the Perspex$^\circledR$ gauging tank. The device employs ‘mass-mode’ gauging to make FDG measurements: the end of the siphon tube is located at a distance *H* below the liquid level in the gauging tank: this generates a fixed pressure difference across the gauging nozzle. Liquid is withdrawn through the nozzle and passes through a mass flowmeter (Rheotherm 100‐L, Intek Inc., Westerville, OH, USA), before being discharged into the holding tank. Liquid is pumped back to the gauging tube by a peristaltic pump (Masterflex L/S; Cole-Parmer, Vernon Hills, IL, USA), and a weir maintains a constant level therein. The holding tank is also temperature-controlled, and allows agitation or pH adjustment. The temperature and pH of the liquid inside the gauging tank is measured using a K-type thermometer and pH electrode, respectively.

The device is controlled via a desktop PC (AMD Athlon, 2 GHz processor, 1 GHz RAM, Windows$^\circledR$ XP) running LabView$^{\mathrm {TM}}$ (version 8.2) control and data collection applications. A calibration program moves the nozzle to a range of $h/d_{\mathrm {t}}$ values and records $\dot{m}$, at a fixed value of *H* (i.e. at fixed $\Delta P_{12}$). All data are stored as a text file, which require post-processing in Microsoft$^\circledR$ Excel$^\circledR$ to extract the calibration constants. A separate LabView$^{\mathrm {TM}}$ module is used to conduct thickness measurements. Measurements are made continuously, and the user defines in advance the position and times at which gauging measurements are made. This allows:

1. Measurements at multiple points on the same sample to evaluate sample homogeneity.
2. Testing of multiple samples under identical experimental conditions.
3. Collection of large quantities of data.

The sFDG device is designed such that the shear stresses that the gauging liquid imposes on the deposit ($\sim$5 to 100 Pa) are comparable to those generated in common cleaning-in-place operations [23]. With water alone, this mechanical force was not sufficient to cause any deformation for the polymerised food lipids encountered in this study. The sFDG device was used predominantly to quantify the effect of cleaning through thickness measurements.

For sFDG thickness measurements, two sets of clearance (*h*) readings were required. The first set, collected during cleaning tests, measured the location of the upper surface of the deposit relative to the fixed position of the displacement sensor, $z_{\mathrm {rel}}^{\mathrm {deposit}}$. The second set measured the location of the substrate surface relative to the same fixed point, $z_{\mathrm {rel}}^{\mathrm {substrate}}$. The thickness of the soil, $\delta$, was then calculated from:

8 $$\begin{aligned} \delta = z_{\mathrm {rel}}^{\mathrm {deposit}} - z_{\mathrm {rel}}^{\mathrm {substrate}} \end{aligned}$$

$z_{\mathrm {rel}}^{\mathrm {substrate}}$ required removal of the sample from the substrate at the end of each test, without changing the position of the substrate. The deposits were typically removed by scraping with a spatula to expose the underlying surface.

Initial sample thickness, $\delta _{0}$, was measured with the sFDG device except in cases where the cleaning agent did not weaken the layers sufficiently to allow measurement of $z_{\mathrm {rel}}^{\mathrm {substrate}}$. In such cases, a vernier caliper was employed to estimate the initial thickness.

#### Cleaning the sFDG Device

Cleaning tests probed the efficacy of various surfactants. In order to minimise contamination by surfactant residues, the following protocol was employed:

1. Rinsing with 10 L of deionised water (20 $^\circ$C).
2. 15 min of pumping deionised water through the device.
3. A final rinse of 10 L of deionised water.

### Contact Angle Measurements

Contact angle measurements were conducted at room temperature using distilled water, diiodomethane, dodecane, hexane, ethylene glycol and formamide using a goniometer contact angle system (SCA 202; DataPhysics Instruments, Filderstadt, Germany). The contact angle was recorded for static sessile drops. Drops approximately 2 $\upmu$m in diameter were syringed onto each surface, with at least 10 repeats for each liquid. The surface energy values for several common probe liquids are listed in Table 5.

### Aims and Objectives

The main objective of this study was to develop and improve understanding of how to clean polymerised soils. More specifically, this can be described as:

1. Developing and characterising a polymerised greasy test soil for study.
2. Testing different cleaning agents using the sFDG device, with water as the solvent.
3. Identifying cleaning mechanisms that lead to the removal of polymerised soils.
4. Evaluating the effect of different surfaces on the cleaning of polymerised soils.

## Results and Discussion

### Solvent Solubility Studies

**Fig. 6:**
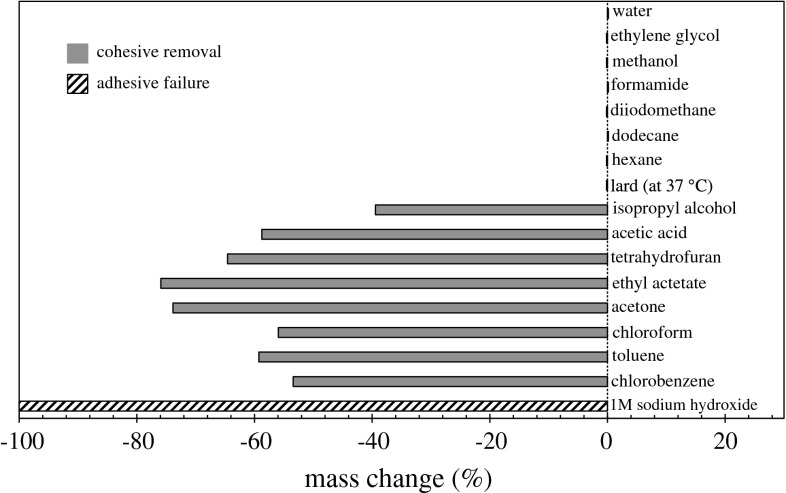
Change in mass of baked lard layers after immersion and agitation in 100 mL of the solvent overnight. All studies conducted at 20 $^\circ$C unless otherwise stated. *Shading* indicates mode of removal

Figure 6 shows the change in mass measured for the baked lard layers immersed in 100 mL solvent overnight. Water, unbaked lard (at 37 $^\circ$C), hexane, dodecane, methanol, ethylene glycol and formamide showed no interaction with these layers. The lack of interaction with unbaked lard confirmed that, for the baked layers, heat treatment had markedly changed the nature of the deposit. These tests also indicated that these solvents were suitable for contact angle measurements. Acetic acid, isopropyl alcohol, ethyl acetate, acetone, tetrahydrofuran, chlorobenzene, toluene and chloroform all caused significant removal (<50 wt% removal). It is not known whether these solvents promoted chemical breakdown of the baked lard.

Separate tests (data not shown here) found that unbaked lard was soluble in chloroform, toluene, benzene and tetrahydrofuran. Baking therefore converted lard, a soft solid material soluble in several common solvents, into a brittle solid deposit, insoluble in the range of solvents tested. As the baked layers were not soluble in any of the test solvents, common analytical tests to study autoxidation, such as gel permeation chromatography, and the peroxide, iodine, *p*-anisidine, thiobarbituric acid and oxirane values could not be used [24].

The 1M sodium hydroxide solution (i.e. pH 14) caused complete adhesive removal of the baked lard deposit. As this proved to be the only substance capable of cleaning the baked lard layers, it was used to clean the SS substrates after testing. No other solvent promoted adhesive removal or led to significant dissolution of the baked layer. These results prompted further tests with different concentrations of NaOH (see below).

**Fig. 7:**
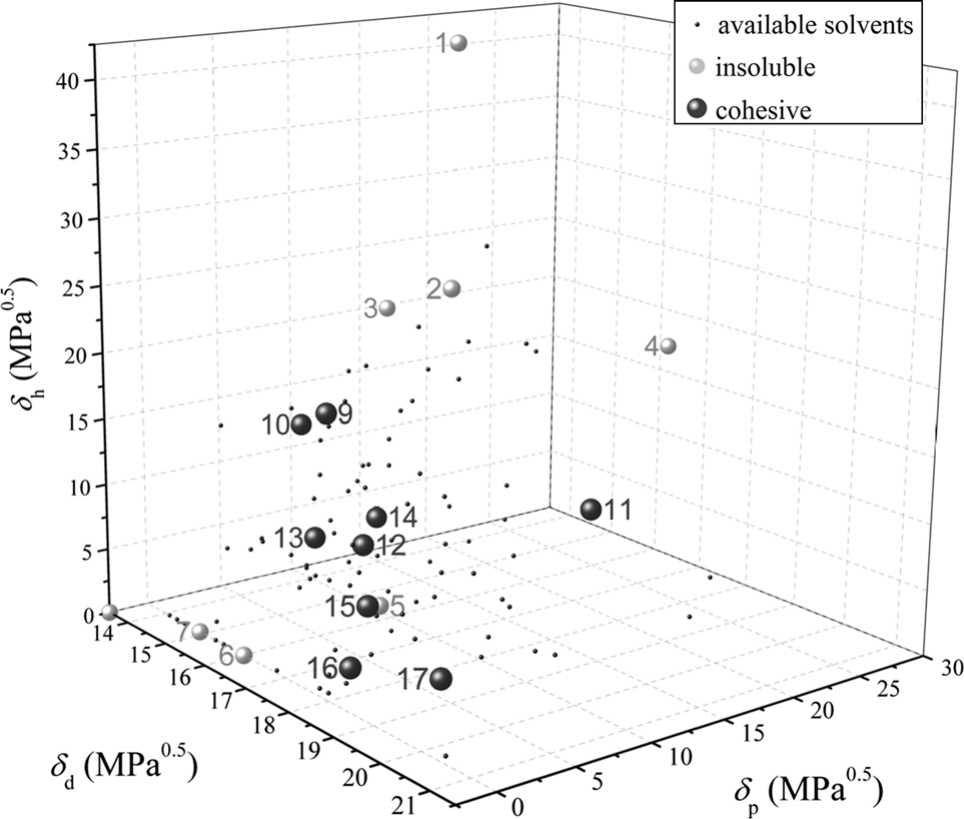
Three-dimensional Hansen solubility parameter diagram summarising the results of the solvent studies in Fig. 6. *Numbers* are solvent labels listed in Table 4. *Grey spheres* denote solvents where there was minimal baked lard-solvent interaction; *brown spheres* show solvents where there was significant cohesive removal (>50 wt%). *The small black dots* indicate solvents with HSPs in the literature

There was no clear trend between the final mass content and any of $\delta _{{\mathrm {d}}}$, $\delta _{\mathrm {p}}$ and $\delta _{\mathrm {h}}$. It was only when the data were presented in three-dimensional plots that a trend became clear, as shown in Fig. 7. The solvents used covered a wide range of available solvents. This plot is *not* a solubility map because the baked lard was *not* soluble in any of the tested solvents. This means that HSP values for the baked layer could not be obtained at 20 $^\circ$C, as the layers were unlikely to dissolve at this temperature. It is noteworthy that the region of interaction is a distance from the location of water (labelled as number 1 on the plot).

### Cleaning Studies

This section discusses sFDG studies on the baked lard samples. All cleaning agents were tested under standard dishwashing conditions, i.e. with water as a solvent, a pH of 10.4 and temperature of 50 $^\circ$C. All tests were conducted for a minimum of 2 h, during which the sFDG device measured the deposit thickness at three separate locations. All tests were repeated at least once. Table 1 lists the surfactants and the concentrations employed in tests. Solution pH was varied with sodium hydroxide. The gauge spent 5 min at each location before changing position. To evaluate the effect of the gauging flow on thickness measurements, one of the three locations was gauged half as often as the other two.

#### Water, pH

As water was the solvent in these studies, the first set of sFDG tests looked at the effect of water on the baked layers, as summarised in Table 2. No visual changes or swelling were observed at pH $\le$10.4. Moreover, the layers were too tough to be removed from the substrate whilst still in the sFDG sample holder: sample thickness was instead measured by vernier calipers before and after testing. At 50 $^\circ$C and pH 11, the FDG measurements indicated an increase in sample thickness, and after approximately 2 h, adhesive removal occurred as large blisters formed underneath the nozzle. The blistered material (see Fig. 1d) was weakly attached to the SS substrate and could easily be removed, usually revealing the underlying substrate to be clean. This type of removal suggested that ingress at the soil–substrate interface caused removal.

**Table 2:** Summary of sFDG tests with lard layers baked on SS and immersed in water

pH	Temperature ($^\circ$C)	$\Delta \delta$ ($\upmu$m)	Absolute thickness measurements	Removal
7.0	19	–	VC	–
7.0	50	–	VC	–
9.5	50	–	VC	–
10.4	19	–	VC	–
10.4	50	–	VC	–
11.0	50	$+$20–30	sFDG	Adhesive

sFDG tests with $H = 100$ mm

*VC* vernier caliper

#### Fairy Liquid

Fairy Liquid was used as a benchmark for standard commercial household detergents. This product contains buffers that maintains a solution pH of 10.4 at 50 $^\circ$C. Immersion in Fairy Liquid solution resulted in no change in sample thickness over the 2-h test.

#### LAS

**Fig. 8:**
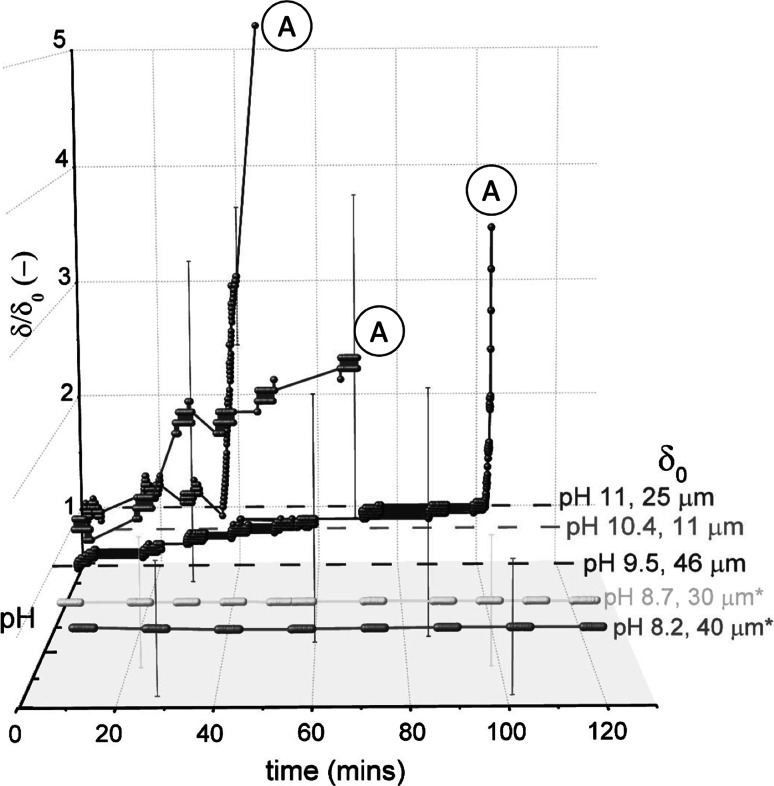
Effect of pH on thickness–time profiles for the baked lard layers immersed in LAS solution (0.88 g per L). *Spheres* denote data points recorded with the sFDG. *Lines* are interpolations of the data. *Dashed lines* on selected plots indicate the baseline of $\delta /\delta _{0}=1.$ *Labels* indicate test pH and $\delta _{0}$. $^*$ in legend indicates that as $\delta _{\mathrm {rel}}^{\mathrm {substrate}}$ could not be measured, $\delta _{0}$ was measured with vernier calipers. Label ‘*A*’ indicates adhesive ‘lift-off’. Test conditions: 50 $^\circ$C, $H=100$ mm. *Error bars* represent measurement uncertainty of $\sim$15 $\upmu$m and are fitted to selected data points only

Figure 8 shows the effect of pH on cleaning with the LAS solution. The data are plotted in terms of $\delta /\delta _{0}$ versus time, where $\delta _{0}$ is the initial sample thickness. There was noticeable variation in $\delta _{0}$, ranging from $\sim$15 to 60 $\upmu$m. At pH values ≤8.7, no swelling or visual changes were observed. At pH 9.5, there was an increase in the sample thickness, $\delta$, over the first 100 min, after which large blisters formed underneath the nozzle and elsewhere across the entire sample. Blister formation occurred more quickly as pH increased. In cases without any surfactant, (adhesive) removal occurred only at pH 11 (see Table 2). Therefore, at pH 9.5 and above, it was the presence of LAS that promoted blister formation. This type of removal is analogous to the roll-up of oily soils. In this case, the ingress of LAS (and associated water molecules) at the soil–substrate interface caused soil rejection (see Fig. 1d). Blisters first arose at the edge of the sample before growing inwards. As the gauging nozzle withdraws liquid into the nozzle, this creates suction and lifts the rejected layer upwards to form large blisters on the deposit.

Gordon et al. [25] used the sFDG device to monitor the extent of swelling. In this study, the occurrence of adhesive removal meant that it was difficult to determine whether swelling occurred or a combination thereof. ‘Lift-off’ occurred when a large part of the sample detached from the substrate, causing a sharp increase in $\delta /\delta _{0}$ on the sFDG plot. Labels ‘A’ in the sFDG plots indicate when ‘lift-off’ occurred.

#### TX-100

**Fig. 9:**
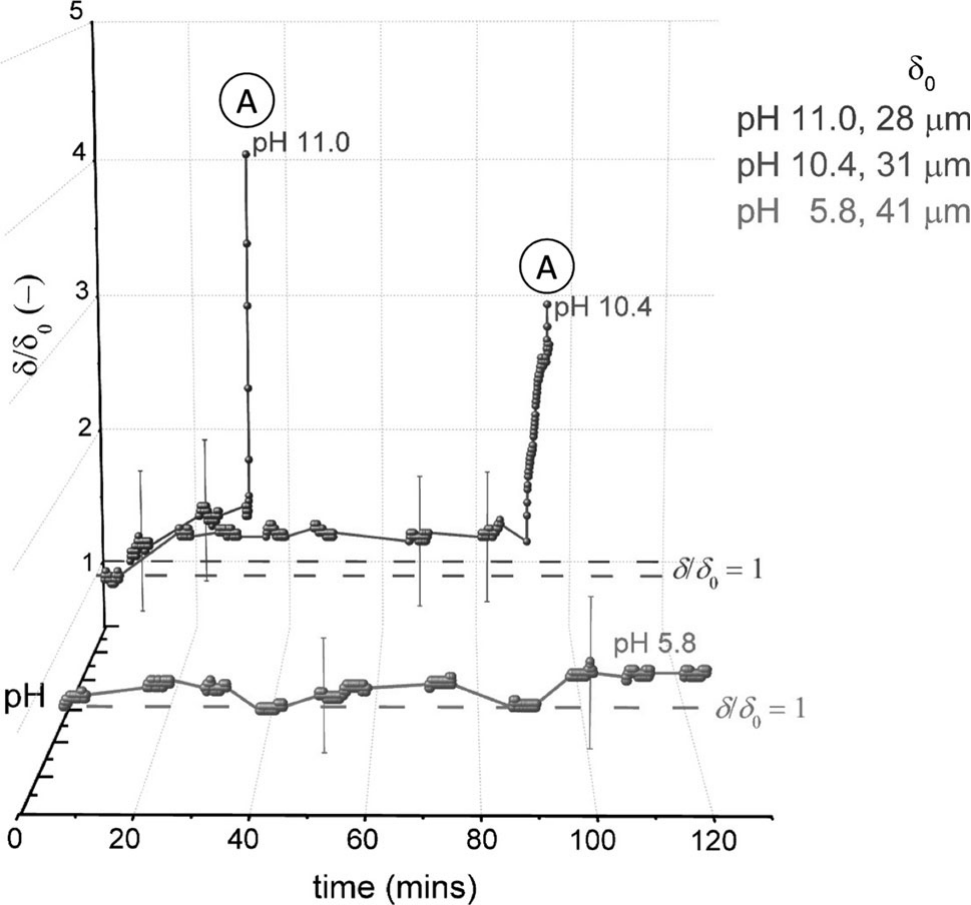
Effect of pH on TX-100 on baked lard layer behaviour at different pH. *Spheres* denote data points recorded with the sFDG. *Lines* are interpolations of the data. *Dashed lines* on selected plots indicate the baseline of $\delta /\delta _{0}=1.$ *Labels* indicate test pH and $\delta _{0}$. ‘*A*’ labels indicates adhesive ‘lift-off’. *Error bars* represent measurement uncertainty of $\sim$15 $\upmu$m and are fitted to selected data points only. Test conditions: 50 $^\circ$C, $H=100$ mm, TX-100 concentration $=$ 0.125 g L$^{-1}$

Figure 9 shows the effect of pH on thickness–time profiles for TX-100 solutions. At pH 5.8, there was no meaningful change in thickness. At pH 10.4, $\delta /\delta _{0}$ increased steadily to $\sim$1.4 over 30 min, suggesting some swelling, before adhesive removal occurred after 90 min. The removal was similar to that observed with LAS. Once the blister was removed, a visually clean substrate remained underneath. As with LAS (Fig. 8), adhesive removal ‘lift-off’ occurred more quickly at pH 11.

#### Silicon-Sealed Edges

The studies with TX-100 and LAS suggested the ingress of surfactants at the soil–substrate interface rather than penetration through the soil at the soil–liquid interface. To test this hypothesis, a layer of silicon grease was coated around the edges of the sample. With LAS and TX-100, there was then no marked change in sample thickness. Moreover, no adhesive removal occurred during the 2-h test. Blocking the surfactants from the soil–substrate interface prevented removal, thus confirming the ingress hypothesis.

#### CTAB

**Fig. 10:**
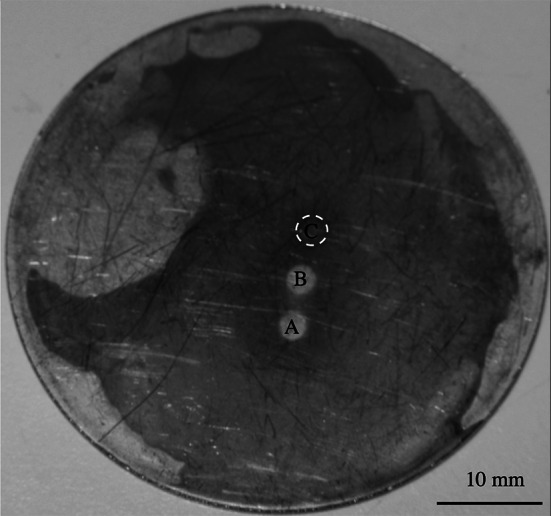
Photograph of a lard layer after 90 min immersion in CTAB at pH 10.4, 50 $^\circ$C, with *H* set as 100 mm. *A*, *B* and *C* mark locations gauged by the sFDG device. *A* and *B* were gauged twice as often as *C*

With CTAB, a different mode of removal was observed. Figure 10 shows a photograph of a baked lard layer following immersion in CTAB for 90 min at pH 10.4 and 50 $^\circ$C. FDG measurements were conducted at the three locations A, B and C. A and B were underneath the gauging nozzle for 35 min each, and the soil at both positions was eroded under the nozzle (instead of large blisters forming), leaving a clean substrate. At location C, for which measurements were made for only 15 min, removal was less complete. This behaviour was observed for all tests with CTAB.

**Fig. 11:**
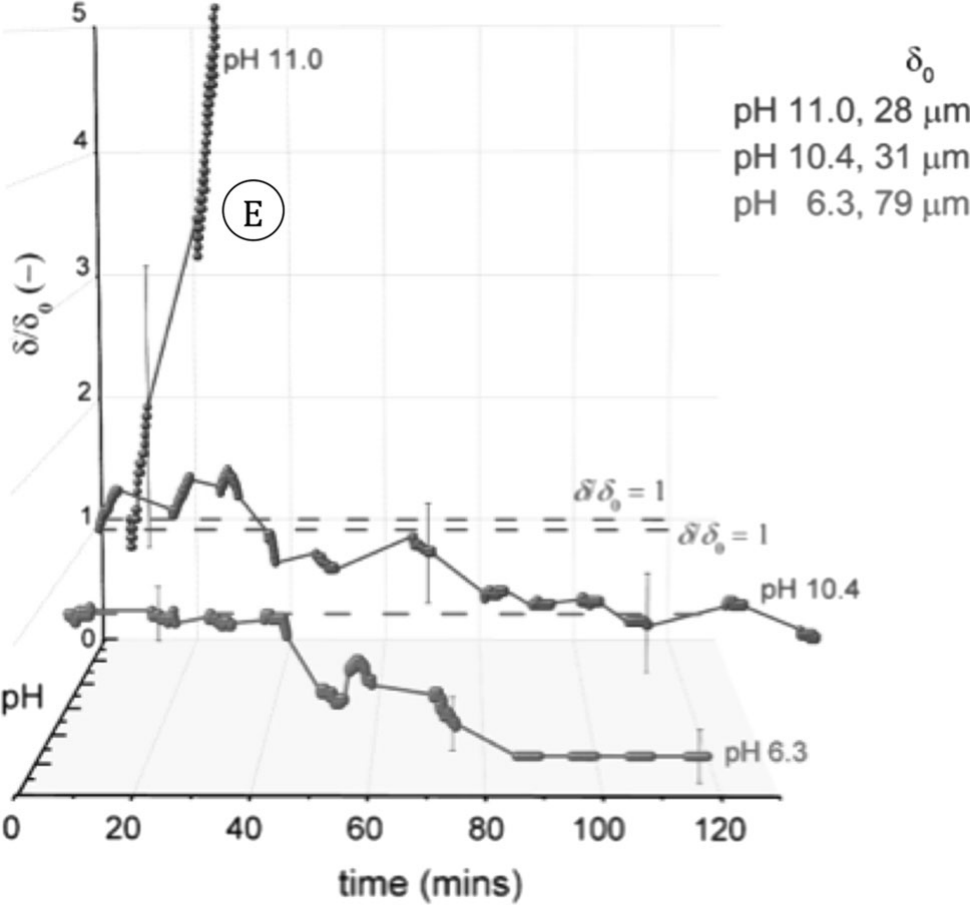
Effect of pH on CTAB cleaning behaviour, with a CTAB concentration of 0.364 g L$^{-1}$. *Spheres* denote data points recorded with the sFDG. *Lines* are interpolations of the data. *Dashed lines* on selected plots indicate the baseline of $\delta /\delta _{0}=1.$ *Labels* denote test pH and $\delta _{0}$. Test conditions: 50 $^\circ$C, $H=100$ mm. *Error bars* represent measurement uncertainty of $\sim$15 $\upmu$m and are fitted to selected data points only. ‘*E*’ indicates erosive removal observed

The $\delta /\delta _{0}$ profiles for the CTAB tests are shown in Fig. 11. At pH 11, the sample undergoes the largest increase in $\delta$, with complete removal occurring after $\sim$50 min. Unlike earlier tests, the removal observed here occurs via cohesive rather than adhesive removal. It is believed that the large increase in $\delta$ is due to spalling debris affecting thickness measurements. At pH 10.4 there is a small initial increase in $\delta$, before sample thickness gradually decreases, suggesting weakening of the layer before erosion occurs. At pH 6.3, no swelling occurs. The same type of removal is seen, however, with a clean surface beneath the nozzle after 85 min.

As removal was only observed directly beneath the nozzle, it is likely that (1) CTAB penetrates the soil at the soil–liquid interface (discussed in more detail later) and (2) some degree of fluid force is required to remove (or erode) the weakened section of deposit. For these reasons, the effect of shear stress was investigated for CTAB. Varying the siphon hydrostatic pressure head, *H* (see Fig. 5), changed the shear stress exerted on the layer, $\tau _{yr}$, in the ring underneath the nozzle lip. $\tau _{yr}$ was estimated using the following expression:

9 $$\begin{aligned} \tau _{yr} = \left( \frac{3 \mu \dot{m} }{ \pi \rho h^2} \right) \frac{1}{r} \end{aligned}$$

Here, *r* is the radial distance from the centre of the nozzle. The shear stress reported is that estimated for $r = 0.5$ mm. This expression has been shown to yield reasonable agreement with the maximum shear stress exerted under the nozzle calculated in CFD simulations [25].

**Fig. 12:**
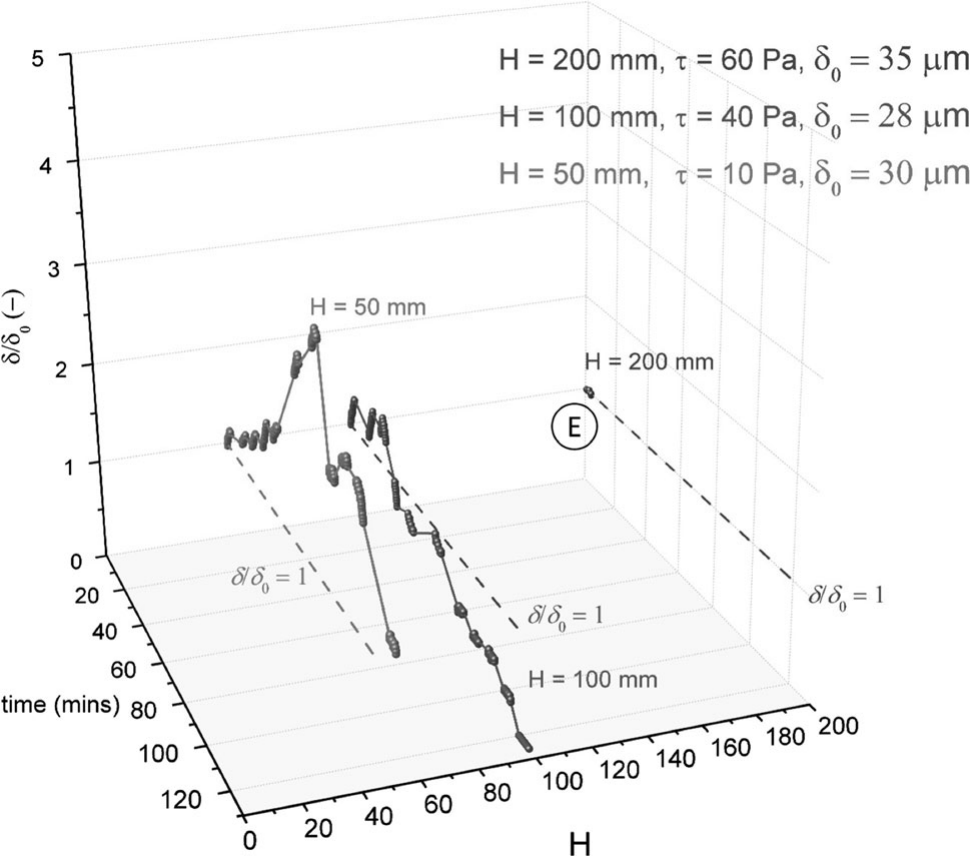
Effect of shear stress (hydraulic forces) on CTAB cleaning tests, with a CTAB concentration 0.364 g L$^{-1}$, 50 $^\circ$C, pH 10.4. *Spheres* denote data points recorded with the sFDG. *Lines* are interpolations of the data. *Dashed lines* on selected plots indicate the baseline of $\delta /\delta _{0}=1.$ *Error bars* represent measurement uncertainty of $\sim$15 $\upmu$m and are fitted to selected data points only. *Legend* indicates siphon head, *H*, estimated $\tau _{yr}$ values during thickness measurements, and $\delta _{0}$. Label ‘*E*’ denotes erosive removal

Figure 12 shows the effect of *H* (and thus $\tau _{yr}$) on the cleaning studies with CTAB. For the lowest value of *H* ($=$50 mm), the lowest shear stress values were exerted on the gauged surface, and this delayed erosive removal from underneath the nozzle. At $H = 50$ mm, the soils swelled more and were subsequently eroded, which is consistent with the layer growing weaker as it swells. Increasing *H* led to faster cohesive removal. At $H = 100$ mm, removal occurred after 90 min, and at $H = 200$ mm, cohesive removal was observed within 5 min. In all cases (i.e. $H = 50 {-} 200$ mm), no removal was observed in the absence of CTAB.

For CTAB blocking, the sample edges with silicon grease did not affect removal behaviour: CTAB instead penetrated the soil at the soil–liquid interface. The removal with CTAB was therefore analogous to the necking mechanism for oily soils (see Fig. 1). There was, however, no discernible trend between ionic strength, surfactant type and removal mechanism.

#### Glass Substrates

Domestic and commercial appliances feature greasy soils baked on a variety of surfaces. Removal via surface ingress is expected to be influenced by the substrate properties, and this was tested here using two different types of borosilicate glass in addition to the SS discs. The first was labelled glass I’ (Edmund Optics$^\circledR$; Barrington, NJ, USA), and the second ‘glass II’ (Soham Scientific, Ely, Cambridgeshire, UK).

Surface energy measurements were conducted on both surfaces, and the results are summarised in Table 3. The fouling behaviour varied across the three substrates. Glass II, which has a contact angle with water, $\theta _{\mathrm{H}_2{\mathrm{O}}}$, similar to that of the stainless steel surface, required 2 h baking at 200 $^\circ$C for the lard to form solid gum-like deposits, compared to 1.5 h at 200 $^\circ$C on the SS discs. The three surface energy components for glass I were very similar to those obtained for the stainless steel. In glass II, however, surface energy data suggest that the larger $\gamma _{\mathrm {SV}}^{\mathrm {AB}}$ and/or lower $\theta _{\mathrm{H}_2{\mathrm{O}}}$ values are related to its long baking time.

**Table 3:** Surface energy and hardening time for lard baked on glass surface

Material	$\theta _{\mathrm{H}_2{\mathrm{O}}}$ ($^\circ$)	$\gamma _{\mathrm {SV}}$ (mJ m$^{-2}$)	$\gamma _{\mathrm {SV}}^{\mathrm {LW}}$ (mJ m$^{-2}$)	$\gamma _{\mathrm {SV}}^{\mathrm {AB}}$ (mJ m$^{-2}$)	Hardening time (h)
Glass I	74.1 ($\pm$3.1)	37.5 ($\pm$0.3)	35.0 ($\pm$0.1)	2.5 ($\pm$0.2)	2.0 ($\pm$0.3)
Glass II	44.6 ($\pm$4.0)	40.4 ($\pm$0.3)	34.8 ($\pm$0.1)	5.6 ($\pm$0.2)	6.0 ($\pm$1.0)
SS 316	71.9 ($\pm$4.4)	37.3 ($\pm$2.0)	35.5 ($\pm$1.8)	2.8 ($\pm$0.3)	1.5 ($\pm$0.3)

Hardening time refers to the time required for the layers to cross the hardening transition when baked at 200 $^\circ$C. $\pm$ denotes standard deviation for contact angle measurements, maximum relative error for the surface energy data, and experimental uncertainty in the hardening time values. The SS results are provided as a reference

**Fig. 13:**
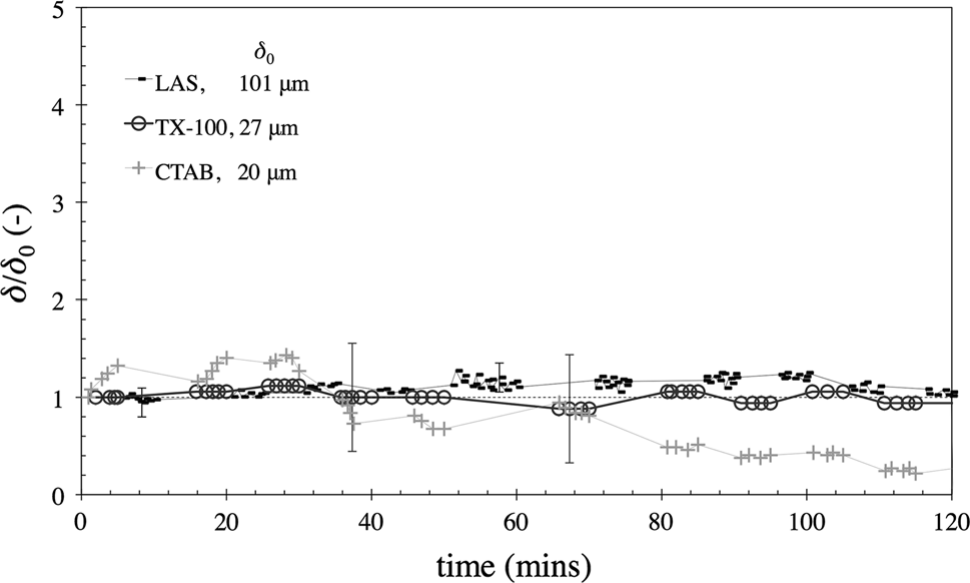
sFDG tests with baked lard layers on glass I. Test conditions: pH 10.4, 50 $^\circ$C, $H=100$ mm. *Error bars* represent measurement uncertainty of $\sim$15 $\upmu$m and are fitted to selected data points only. *Lines* are interpolations of the data. Some data points are removed to improve clarity. *Legend* indicates $\delta _{0}$. Abscissa scale matches other plots

Figure 13 shows sFDG data for cleaning tests with glass substrates. For LAS, TX-100 and CTAB on glass I, no substantial change in thickness is evident. It appears that ingress of these surfactants at the soil-substrate interface did not occur in a similar manner to that of stainless steel. This could be due to the nature of the glass–soil interface and/or the nature of the baked lard, which had been baked for a further 30 min on this type of glass. Cleaning tests with TX-100 and LAS were conducted with the lard layers baked on SS for 2 h (instead of the usual 1.5 h baking time). In both cases, there was a small increase in $\delta$ (20–30 $\upmu$m, data not shown). For TX-100, no adhesive removal (or ‘lift-off’) occurred during the 2-h test. For LAS, small blisters were observed at the edge of the baked deposit after 1 h 50 min, and adhesive ‘lift-off’ occurred at the centre of the disc after 2 h 5 min (data not shown), thus confirming that the additional 30 min baking time created layers which were more difficult to remove. This is in agreement with the study by Ali et al. [26], which found that longer baking times led to tougher layers.

#### Surfactant Mixtures

**Fig. 14:**
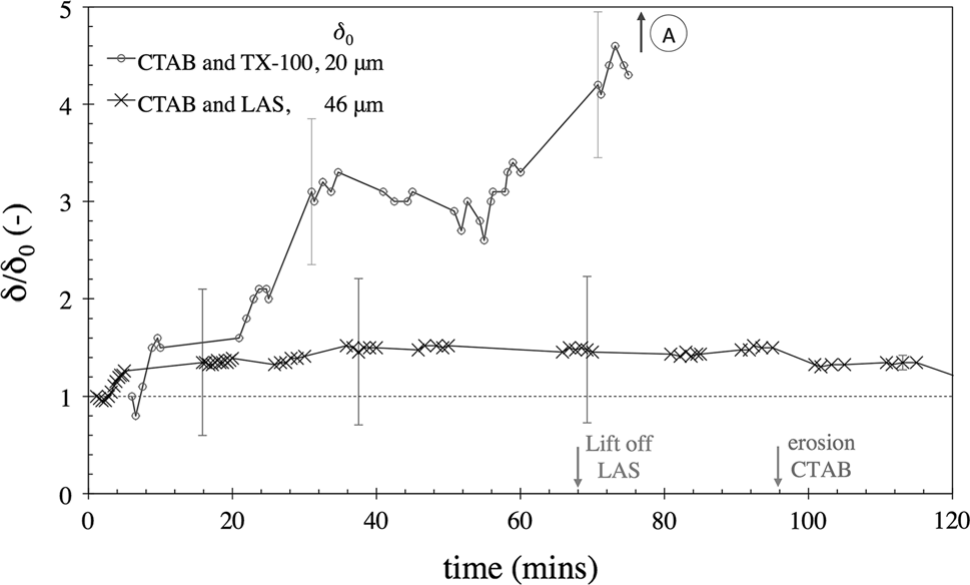
sFDG tests with CTAB-LAS and CTAB-TX-100 mixtures. Lard layers baked on SS. Test conditions: pH 10.4, 50 $^\circ$C, $H=100$ mm. *Error bars* represent measurement uncertainty of $\sim$15 $\upmu$m and are fitted to selected data points only. *Lines* are interpolations of the data. Some data points are removed to improve clarity. *Arrow* indicates that adhesive ‘lift-off’ occurred. *Label A* indicates where adhesive lift off occurred. *Arrows* on time axis indicate where removal occurred with LAS and CTAB in earlier figures

It is common for commercial cleaning agents to contain a combination of surfactants. As removal with CTAB differed from that observed with LAS and TX-100, mixtures of CTAB-LAS and CTAB-TX-100 were studied. Figure 14 shows the $\delta /\delta _{0}$ profiles for these surfactant blends. For the single-surfactant cleaning solutions, LAS and TX-100 promoted ‘lift-off’ after 70 and 90 min, respectively. CTAB gave rise to erosive removal after 90 min. With the CTAB-LAS solution, there was a small amount of swelling and no removal, indicating surfactant antagonism. For the CTAB and TX-100 solution, however, adhesive ‘lift-off’ occurred after 80 minutes, which was similar to the results with TX-100 alone (see Fig. 9). As no erosion was observed underneath the nozzle prior to blister formation (as would be expected), it appears that TX-100 also inhibited the CTAB erosive process. The reason that CTAB did not inhibit the TX-100 ingress requires further investigation.

## Summary

Baked lard layers were prepared as models of oxidised oil soils. Solvent solubility studies were conducted on the baked lard layer to identify which common analytical techniques, if any, could be used to study the baked soils. The test soils, however, were not soluble in any of the solvents used, precluding the use of many standard chemical analysis techniques.

The scanning FDG device was used in cleaning studies monitoring layer thickness while the soil was in contact with the cleaning solution. There was no visible interaction with or measurable change with water at pH 7–10.4, and 19–50 $^\circ$C. A commercial dishwasher solution (Fairy Liquid and 50 $^\circ$C), also at pH 10.4, caused no change in deposit thickness. Solution pH was important: at pH 11, 50 $^\circ$C adhesive removal occurred rapidly. The faster removal at higher pH may be related to the charge effects on the soil layer, substrate and cleaning agent, but this requires further investigation.

Cleaning tests with surfactants identified two mechanisms for removal of these polymerised lipid soils, as summarised in Fig. 1. Surfactants LAS and TX-100 promoted removal by interrupting adhesive bonding or ‘lift-off’, whereby large blisters formed on the SS surface. The blistered material was then sucked upwards by the gauging flow and was readily removed from the substrate to leave a clean SS surface. It was hypothesised that these cleaning agents achieved adhesive removal by ingress at the soil–substrate interface. Subsequent tests with sealed edges inhibited adhesive removal, confirming the ingress hypothesis. The two removal mechanisms were analogous to the roll-up and necking of oily soils.

CTAB, a cationic surfactant, promoted cleaning by penetrating through the soil at the soil–liquid interface and weakening cohesive interactions within the layer. Soil removal was only observed directly underneath the gauging nozzle, where the shear stresses exerted on the deposit by the gauging flow were sufficient to cause removal. Increasing the shear stress exerted by the nozzle by increasing *H* resulted in markedly quicker removal.

Tests on glass substrates were not directly comparable, as the lard had to be cooked for a longer time in order to generate a ‘hard’ deposit. The CTAB cleaning action, involving penetration of the soil from the soil–liquid interface, was not affected by the substrate, as expected. The ‘lift-off’ mechanism promoted by LAS and TX-100 is controlled by the state of the soil/substrate/solution contact line; tests with glass and stainless steel substrates highlighted this. Whereas LAS and TX-100 both promoted lift in soils baked on SS for 90 min, extending the baking time on SS by 30 min prevented lift off at the soil edges for both surfactants. The glass substrates required longer baking times, which suggests that the autoxidation kinetics differed, either due to differences in heat transfer or initiation rates. The soil generated on glass I after 120 min was not amenable to lift-off with TX-100, but was observed, albeit delayed, with LAS.

Tests with mixtures of CTAB-LAS and CTAB-TX-100 showed inhibition of CTAB action. TX-100 was not inhibited by CTAB. LAS, which is commonly used in commercial cleaning agents, was inhibited by CTAB.

## Appendix

See Tables 4 and 5.

**Table 4:** Hansen solubility parameters for solvents used in this study, recorded at 20 $^\circ$C unless otherwise stated

Solvent	Reference number	Hansen solubility parameters		
		Nonpolar interaction, $\delta _{\mathrm {d}}$ (MPa$^{0.5}$)	Polar interaction, $\delta _{\mathrm {p}}$ (MPa$^{0.5}$)	Hydrogen bonding, $\delta _{\mathrm {h}}$ (MPa$^{0.5}$)
Water*	1	15.5	16.0	42.0
Ethylene glycol*	2	17.0	11.0	26.0
Methanol*	3	15.1	12.3	22.3
Formamide*	4	17.2	26.2	19.0
Diiodomethane*	5	17.8	3.9	5.5
Dodecane*	6	16.0	0.0	0.0
Hexane*	7	14.9	0.0	0.0
Lard^†^	8	15.9	1.2	5.4
Isopropyl alcohol	9	15.8	6.1	16.4
Acetic acid	10	14.5	8.0	13.5
Dimethyl sulfoxide	11	18.4	16.4	10.2
Tetrahydrofuran	12	16.8	5.7	8.0
Ethyl acetate	13	15.8	5.3	7.2
Acetone	14	15.5	10.4	7.0
Chloroform	15	17.8	3.1	5.7
Toluene	16	18.0	1.4	2.0
Chlorobenzene	17	19.0	4.3	2.0
Sodium hydroxide$^\ddag$	18	–	–	–

Source: [13]

Reference numbers are added to aid the reader in interpreting HSP plots shown later

* Used in contact angle measurements

$$^\dagger $$ 37 $$^\circ $$C

$^\ddag$ No HSP value available

**Table 5:** Values of surface energy components for contact angle probe liquids (in mJ m$^{-2}$)

Liquid	$\gamma _{\mathrm {LV}}$	$\gamma _{\mathrm {LV}}^{\mathrm {LW}}$	$\gamma _{\mathrm {LV}}^{\mathrm {AB}}$	$\gamma _{\mathrm {LV}}^+$	$\gamma _{\mathrm {LV}}^-$	References
Water	72.8	21.8	51.0	25.5	25.5	[27]
formamide	58.0	39.50	19.0	2.28	39.6	[27]
Diiodomethane	50.8	50.8	$\approx$0	$\approx$0	0	[28]
Ethylene glycol	48.0	29.0	19.0	1.92	47.0	[27]
Dodecane	25.4	25.4	0	0	0	[29]

## Roman

$$C_{\mathrm {d}}$$: Discharge coefficient (–)
*d*: Inner diameter of gauging tube (m)
$d_{\mathrm {t}}$: Inner diameter of nozzle (m)
*H*: Siphon hydrostatic pressure head (m)
$\Delta H_{\mathrm {vap}}$: Enthalpy of vaporisation (J mol$^{-1}$)
*h*: Clearance between nozzle and gauged surface (m)
$h_{\mathrm {o}}$: Clearance between nozzle and substrate (m)
$\Delta P_{12}$: Pressure drop across the gauging nozzle (Pa)
$\dot{m}$: Mass flow rate (kg s$^{-1}$)
*r*: Radial coordinate (m)
$V_{\mathrm {m}}$: Molar volume (m$^{3}$ mol$^{-1}$)

## Greek

$\delta$: Thickness of deposit layer (m)
$\delta _{\mathrm {d}}$: Dispersive solubility parameter (MPa$^{0.5}$)
$\delta _{\mathrm {h}}$: Hydrogen bonding solubility parameter (MPa$^{0.5}$)
$\delta _{{i}}$: Solubility parameter for species *i* (MPa$^{0.5}$)
$\delta _0$: Initial sample thickness (m)
$\delta _{\mathrm {p}}$: Polar solubility parameter (MPa$^{0.5}$)
$z_{\mathrm {rel}}^{\mathrm {deposit}}$: Nozzle clearance relative to deposit (m)
$z_{\mathrm {rel}}^{\mathrm {substrate}}$: Nozzle clearance relative to substrate (m)
$\gamma _{ij}$: Surface energy between phases *i* and *j* (J m$^{-2}$)
$\gamma _{ij}^+$: Electron acceptor component of surface energy between phases *i* and *j* (J m$^{-2}$)
$\gamma _{ij}^-$: Electron donor component of surface energy between phases *i* and *j* (J m$^{-2}$)
$\gamma _{ij}^{\mathrm {AB}}$: Acid-base component of surface energy between phases *i* and *j* (J m$^{-2}$)
$\gamma _{ij}^{\mathrm {LW}}$: Lifshitz–van der Waals component of surface energy between phases *i* and *j* (J m$^{-2}$)
$$\kappa $$: Lip width (m)
$\Lambda$: Nozzle entry length (m)
$\mu$: Fluid viscosity (Pa s)
$\rho$: Fluid density (kg m$^{-3}$)
$\tau _{yr}$: Wall shear stress on *y*-plane in the *r*-direction (Pa)
$\theta$: Equilibrium contact angle ($^\circ$)
$\theta _{\mathrm{H}_2\mathrm{O}}$: Equilibrium contact angle with water ($^\circ$)
$\varphi$: Internal nozzle angle ($^\circ$)

## Abbrevations

CMC: Critical micelle concentration
CTAB: Cetyl trimethyl ammonium bromide
FDG: Fluid dynamic gauging
HSP: Hansen solubility parameter
LAS: Linear alkylbenzene sulfonic acid
NaOH: Sodium hydroxide
sFDG: Scanning fluid dynamic gauge
SS: Stainless steel
TX-100: Triton X-100
VC: Vernier caliper
